# Global Research Trends in Extracellular Vesicle–Based Therapy for Regenerative Medicine: A Bibliometric Analysis (2014–2024)

**DOI:** 10.3390/bioengineering13020247

**Published:** 2026-02-20

**Authors:** Ramya Lakshmi Rajendran, Atharva Anand Mahajan, Sathish Muthu, Sathish Kumar Rajappan Chandra, Prakash Gangadaran, Byeong-Cheol Ahn

**Affiliations:** 1 BK21 FOUR KNU Convergence Educational Program of Biomedical Sciences for Creative Future Talents, Department of Biomedical Sciences, School of Medicine, Kyungpook National University, Daegu 41944, Republic of Korea; ramyag@knu.ac.kr; 2 Department of Nuclear Medicine, School of Medicine, Kyungpook National University, Daegu 41944, Republic of Korea; 3 Cardiovascular Research Institute, Kyungpook National University, Daegu 41944, Republic of Korea; 4 Advanced Centre for Treatment, Research and Education in Cancer, Navi Mumbai 410210, Maharashtra, India; atharva66mahajan@gmail.com; 5 Orthopaedic Research Group, Department of Orthopaedics, Coimbatore 641005, Tamil Nadu, India; 6 Central Research Laboratory, Meenakshi Medical College Hospital and Research Institute, Meenakshi Academy of Higher Education and Research, Kanchipuram 631552, Tamil Nadu, India; 7 Clinical Trial & Research Unit, Interdisciplinary Institute of Indian System of Medicine (IIISM), SRM Institute of Science and Technology, Kattankulathur 603203, Tamil Nadu, India; ayursatish@gmail.com; 8 Department of Nuclear Medicine, School of Medicine, Kyungpook National University Hospital, Daegu 41944, Republic of Korea

**Keywords:** extracellular vesicles, regenerative medicine, scientometric (bibliometric) analysis, exosomes

## Abstract

Background: Extracellular vesicles (EVs) have emerged as promising cell-free therapeutic agents in regenerative medicine due to their ability to deliver bioactive molecules with enhanced stability and low immunogenicity. Their potential to replicate stem cell functions without the risks of live-cell transplantation has catalyzed a surge in global research. Objective: This study aims to perform a scientometric analysis of EV-based regenerative medicine research from 2014 to 2024, identifying publication trends, influential contributors, thematic clusters, and translational challenges. Methods: Data were retrieved from the Web of Science Core Collection and analyzed using CiteSpace software. The analysis included journal impact mapping, co-authorship networks, co-citation analysis, and thematic cluster identification. Metrics such as citation bursts, total link strength, and silhouette values were used to assess influence and thematic coherence. Results: The most prolific journals were Stem Cell Research & Therapy and International Journal of Molecular Sciences. China led in publication volume, while the USA dominated citation impact. Foundational works by Théry and Lai, including the MISEV guidelines, shaped methodological standards. Nine thematic clusters were identified, including oxidative stress, small EVs, mesenchymal stromal cells, muscle regeneration, and chronic kidney disease. A strategic shift toward engineered EVs and novel sources such as iPSCs and macrophages was evident. Key translational barriers include lack of standardization, scalability issues, and regulatory ambiguity. Conclusions: EV-based therapies are transitioning from foundational research to clinical application. Overcoming methodological and regulatory challenges will be critical to realizing their full therapeutic potential in regenerative medicine.

## 1. Introduction

In recent decades, the field of regenerative medicine has shifted its focus from whole-cell therapies to cell-free alternatives, with extracellular vesicles (EVs) emerging as a promising new approach [1,2,3]. EVs are a heterogeneous population of nanosized particles secreted by cells that act as natural delivery vehicles, carrying a diverse cargo of proteins, lipids, and nucleic acids from their parent cells to recipient cells [4,5]. This intercellular communication allows them to orchestrate a wide range of biological processes, including tissue repair, immune regulation, and angiogenesis [6], while avoiding the risks associated with live-cell transplantation, such as immune rejection and tumorigenic potential [7].

The therapeutic potential of EVs, particularly those derived from mesenchymal stem cells (MSCs), has been demonstrated in numerous preclinical studies for a variety of conditions, including kidney and cardiovascular diseases [8]. EVs offer several advantages over traditional stem cell therapies, including enhanced stability, low immunogenicity, and the ability to be stored for extended periods [8].

Despite this promise, the translation of EV-based therapies into widespread clinical application faces significant challenges. These include the lack of standardized isolation and characterization methods, scalability issues for mass production, and the need for a better understanding of EV biodistribution and pharmacokinetics [9]. Given the rapid advancement of this field [10], a comprehensive scientometric analysis is essential to understand the current research landscape, identify key trends, and pinpoint future research directions. Despite the rapid expansion of EV research, there has been no consolidated bibliometric synthesis on this domain, and this study addresses this gap by analyzing publications from 2014 through 2024. This ensures that the most recent developments are captured and provides a comprehensive overview of global research trends and translational challenges. This study aims to provide a detailed overview of the progress and challenges in EV-based regenerative medicine from 2014 to 2024 through a systematic analysis of scientific publications.

## 2. Methodology

The study employs a scientometric analysis to systematically and quantitatively analyze the research landscape of EV-based therapy for regenerative medicine. This method uses various metrics to evaluate research trends, collaboration patterns, and influential publications.

### 2.1. Data Source

The primary data source for this analysis was the Web of Science (WoS) Core Collection, selected for its structured citation indexing and compatibility with CiteSpace software (version 6.3.R1 Basic). The search was conducted using the Boolean string: (“extracellular vesicles” OR “exosomes”) AND “regenerative medicine”. The timespan was set from January 2014 to December 2024. Only peer-reviewed articles and reviews published in English were included. Conference abstracts, editorials, letters, and non-English documents were excluded. The search was conducted on 10 September 2025. Duplicate records were removed prior to analysis to ensure accuracy and reproducibility. Although Scopus and PubMed were considered, WoS was chosen due to its standardized metadata and citation network features, which are essential for co-citation and clustering analysis. Scopus offers broader coverage but less consistency in citation formatting, while PubMed, although strong in biomedical literature, lacks citation network data required for scientometric mapping. Future studies may benefit from triangulating across multiple databases to enhance coverage.

### 2.2. Data Visualization and Analysis

The main tool used for data visualization and analysis was CiteSpace, a software application designed to identify and visualize trends in scientific literature along with VOSViewer. All analyses were performed using CiteSpace v.6.3.R1 Basic (Free Version). Default settings were applied without modification. The parameters included a g-index (k = 25), Look-back Retrospective Factor (LRF) = 2.5, Look-back per Node (L/N) = 10, Look-back Years (LBY) = 5, e = 1.0, with a timespan of 2014–2024 (slice length = 1 year). No pruning was applied. We used VOSViewer version 1.6.20 to generate certain network visualization figures. The analysis included:

- **Journal Analysis**: Evaluating the importance and influence of journals based on the number of highly cited articles they published.
- **Scientific Co-operation Network Analysis**: Mapping co-occurrence networks among authors, institutions, and countries to show collaboration patterns.
- **Co-citation Analysis**: Identifying the most influential articles by analyzing how often they are cited together.
- **Cluster Analysis**: Grouping research into thematic clusters to identify distinct research domains and hotspots.

The size of the nodes in the generated knowledge maps indicates the frequency of occurrence, while the links represent the connections between them, such as co-authorship or co-citation. The silhouette value was used to measure the homogeneity of the clusters, with a value closer to 1 indicating higher homogeneity and reliability. The analysis also identified citation bursts, which indicate a sudden increase in the citation frequency of an article, highlighting its impact and influence on the field during a specific period. The raw data analyzed in this study are available in the Supplementary Files.

## 3. Results

### 3.1. Journal Analysis

**Figure 1:**
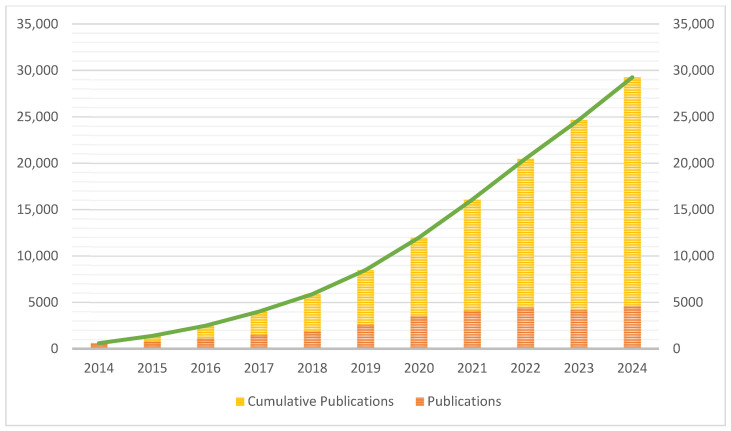
Publication trend in the field of regenerative medicine pertaining to exosomes. Annual publications (orange bars) and cumulative publications (yellow stacked bars) from 2014 to 2024. The green line represents the overall growth trend in cumulative publications over time, highlighting a steady and accelerated increase in research output in recent years.

The field of EV-based therapy for regenerative medicine is experiencing rapid growth, which is reflected in the scientometric analysis of publications from 2014 to 2024 as shown in Figure 1. A key aspect of this analysis is understanding which journals are at the forefront of this research. The data provided, including total documents, citations, and total link strength, paints a clear picture of the most influential journals in the field.

**Figure 2:**
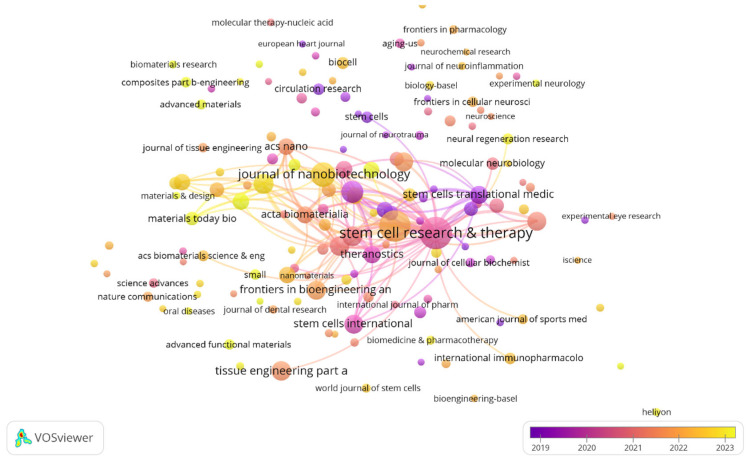
Journal citation network of extracellular vesicle-based therapy for regenerative medicine. Node size reflects publication frequency, while link thickness indicates the strength of relationships between journals. Colors represent the average publication year (blue/purple = earlier; yellow = more recent). VOSviewer: Visualization of Similarities Viewer.

The dominance of *Stem Cell Research & Therapy* (134 documents) and *International Journal of Molecular Sciences* (121 documents) indicates that the primary audience for EV-based regenerative medicine remains rooted in the stem cell and molecular biology communities as shown in the Figure 2. This suggests that EVs are currently being validated as the mechanistic ‘proxies’ for traditional cell therapies rather than as a standalone pharmacological class.

Other significant journals include the “Journal of Nanobiotechnology” (73 documents, 35,354 link strength) and “Scientific Reports” (59 documents, 33,604 link strength). Specialized journals like the “Journal of Extracellular Vesicles” are also highly influential, boasting a high citation count despite a lower document number, which highlights its role in publishing impactful, high-quality research. The network visualization also illustrates these connections, with journals like “Theranostics” and “Biomaterials” forming a tightly linked cluster, emphasizing the interdisciplinary nature of the field where EVs are studied as both therapeutic agents and drug delivery systems. The growing number of publications across these diverse journals reflects the field’s maturity and its integration into a wide array of research disciplines [11].

This rapid expansion is further supported by the increase in publications across various subfields, such as oncology, degenerative musculoskeletal diseases, and drug delivery, which often have their own leading journals. The consistent rise in research output from 2014 to 2024 indicates that scientists are increasingly focusing on the therapeutic potential of EVs as a promising alternative to traditional stem cell therapies, which have been used to treat conditions like diabetes [12]. This shift is driven by the advantages of EVs, including their enhanced stability, lower manufacturing costs, and reduced immunogenicity. The consistent increase in research output and the emergence of these key journals indicate a strong and growing academic interest in EVs as a viable option for regenerative medicine. The top 15 journals publishing highly cited research works on EV-based therapeutics in regenerative medicine is listed in Table 1.

**Figure 3:**
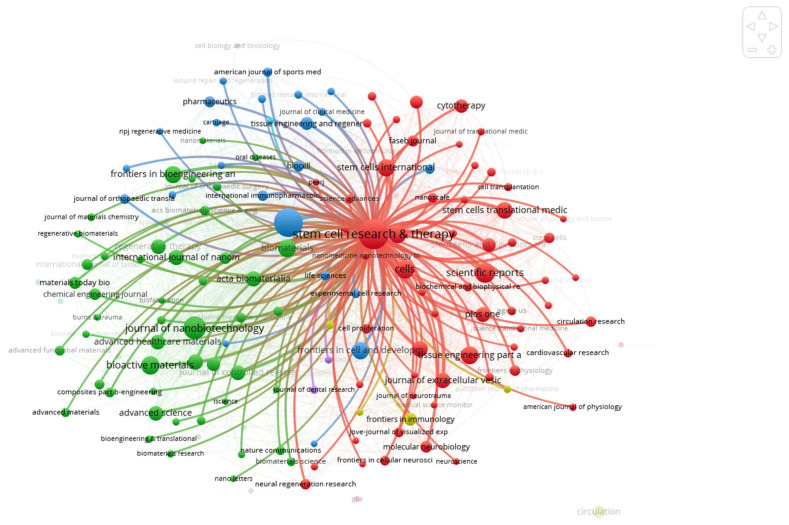
Journal citation network strength of extracellular vesicle-based therapy for regenerative medicine. Node size represents citation frequency, while link thickness indicates the strength of co-citation relationships.

Citations indicate total citations of the identified papers, and Total Link Strength reflects the degree of co-authorship and co-citation connections among journals. The prominence of *Stem Cell Research & Therapy* and *International Journal of Molecular Sciences* as central hubs indicates that EV research is currently being framed as a direct extension of stem cell biology rather than a standalone field. The high “link strength” of these journals suggests that they are the primary gatekeepers for validating the paracrine mechanisms of regenerative medicine. The dominance of *Stem Cell Research & Therapy* and *International Journal of Molecular Sciences* suggests that EV research is currently consolidated within the broader framework of stem cell biology, where vesicles are viewed as the primary functional units of cell-free therapy as shown in Figure 3.

### 3.2. Scientific Co-Operation Network Analysis

**Figure 4:**
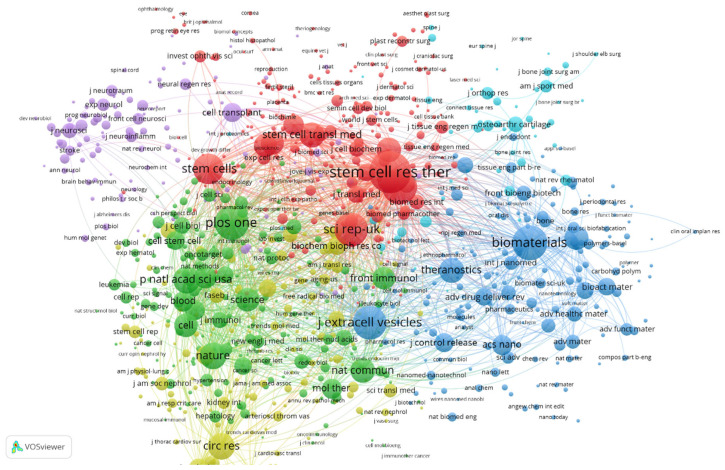
Network of co-citation of journals of extracellular vesicle-based therapy for regenerative medicine. Node size corresponds to co-citation volume, and link thickness reflects the strength of co-citation relationships.

The collaborative landscape of EV-based therapy for regenerative medicine is characterized by a high degree of multidisciplinary synergy, as visualized in the bibliometric network map in Figure 4. This analysis reveals a highly interconnected ecosystem where journals are categorized into distinct thematic clusters, reflecting the field’s intellectual diversity and the convergence of various scientific domains.

The Red Cluster, dominated by journals such as *Stem Cell Research & Therapy* and *Stem Cell Translational Medicine*, serves as the translational hub, reinforcing the role of EVs as functional extensions of stem cell therapy. Parallel to this, the Blue Cluster, anchored by *Biomaterials*, *ACS Nano*, and *Journal of Controlled Release*, highlights a dominant trend in bioengineering and nanotechnology, focusing on the development of “engineered” vesicles and sophisticated delivery platforms. The central positioning of multidisciplinary giants like *Nature*, *Science*, and *PLoS ONE* within the Green Cluster indicates that EV research has transitioned from a niche interest to a foundational pillar of modern biological science, integrated deeply with immunology and molecular biology. Furthermore, the emergence of specialized clusters in neurology and orthopedics, represented by journals such as *Stroke* and *Osteoarthritis and Cartilage*, demonstrates the successful migration of EV therapeutics into organ-specific clinical applications.

The high total link strength and close proximity between the biomaterials and stem cell clusters in Figure 4 suggest that the most impactful research currently occurs at the intersection of material science and regenerative biology. Ultimately, this robust co-operation network illustrates a decade of evolution where the convergence of diverse disciplines is actively overcoming hurdles in EV manufacturing and standardization, paving the way for the next generation of cell-free regenerative medicine.

### 3.3. Co-Citation Analysis

Co-citation analysis, a method for mapping the intellectual structure of a scientific field, reveals the most influential authors, documents, and journals by tracking how often they are cited together in other publications. This analysis for EV-based regenerative medicine from 2014 to 2024 shows a clear hierarchy of key contributors and foundational research.

The data highlights several leading authors with high citation counts and strong co-citation links. The massive citation volume for Théry (658) and Lai (509) reflects a field that is still heavily reliant on foundational protocols and biogenesis definitions as shown in Table 2. The central position of the MISEV (Minimal Information for Studies of Extracellular Vesicles) guidelines in the co-citation network highlights that standardization is not just a trend, but the intellectual core around which all therapeutic applications are built.

**Figure 5:**
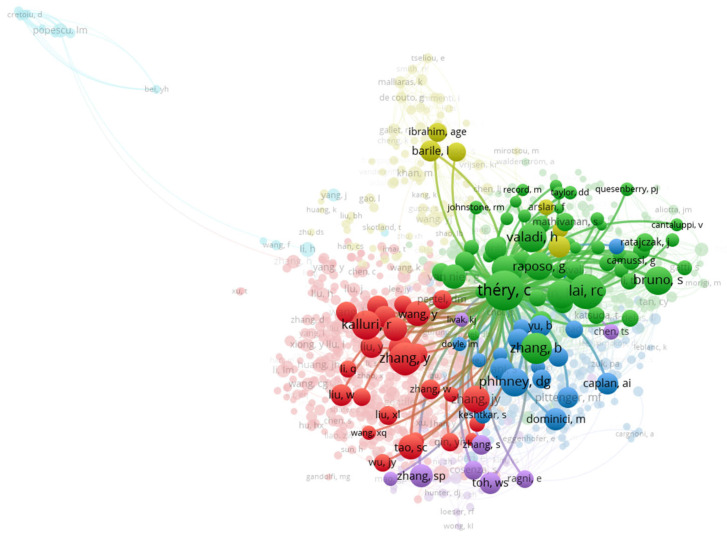
Network of cocitation of the author of extracellular vesicle-based therapy for regenerative medicine. Each node represents an individual author, with node size proportional to publication output. Link thickness indicates the strength of cocitation.

When analyzing the cited documents, the most influential papers are those that established key concepts and methodologies. A paper by R.C. Lai et al. [13] from 2010 on the proteome of MSC-derived exosomes has been highly cited, indicating its foundational role in the field. Another key reference is by C. Théry et al. [14] from 2018 (Figure 5), which established the MISEV guidelines, a critical step toward standardizing EV research protocols. These guidelines have been essential for improving the rigor and reproducibility of studies on EV biogenesis, function, and therapeutic potential. The high citation counts for papers by Ibrahim (2014) [15] and Zhang (2018) [16] further indicate their importance in shaping the intellectual landscape of the field.

Journal co-citation analysis provides further insight into the field’s intellectual core. “Stem Cell Research & Therapy” and “Biomaterials” are at the center of the co-citation network, with the highest total link strength, reflecting their role in publishing core literature that is frequently cited alongside other foundational works. This shows a strong link between EV-based research and the fields of cell therapy and biomaterials, emphasizing the interdisciplinary nature of regenerative medicine. The clusters formed by these journals represent the intellectual communities that are driving the field forward. The high co-citation frequency of the MISEV2018 guidelines by Théry et al. underscores the field’s urgent move toward methodological rigor and standardization, which is a prerequisite for any clinical transition in regenerative medicine.

The international cooperation network reveals a “hub-and-spoke” model of research. While the USA acts as a central intellectual hub—leading in citation impact and foundational methodology (H-index)—China serves as the primary engine for publication volume and preclinical testing. The links between these nations suggest a high degree of knowledge transfer, particularly regarding the translation of MSC research into clinical-grade EV applications.

### 3.4. Cluster Analysis

**Figure 6:**
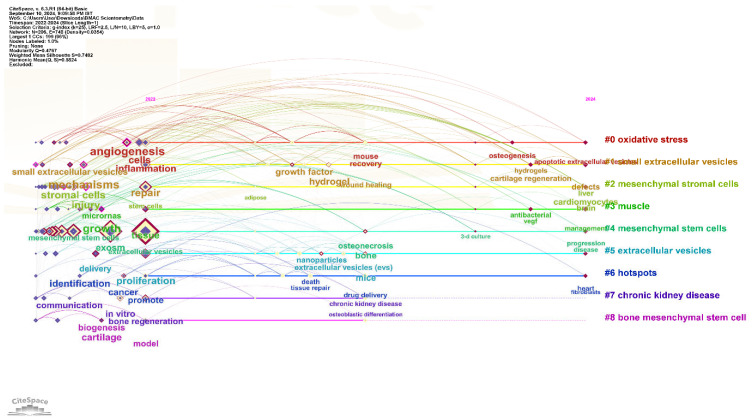
Timeline summary of the various clusters and their interactions on extracellular vesicle-based therapy for regenerative medicine. Each horizontal line represents a thematic cluster. EVs: Extracellular Vesicles; MSCs: Mesenchymal Stem Cells.

The cluster analysis reveals distinct, yet interconnected, research domains within the field of EV-based therapy for regenerative medicine. Each cluster represents a specific area of focus, with highly reliable silhouette values demonstrating the homogeneity of the topics within each group as shown in Figure 6.

#### 3.4.1. Cluster #0 Oxidative Stress

This important research cluster focuses on the role of EVs in mitigating oxidative stress, a central contributor to tissue damage and aging. Studies—including the highly cited review by Chiaradia et al. (2021) [17]—demonstrate that oxidative stress leads to changes in the amount and molecular cargo of released EVs. These EVs can carry both harmful oxidized molecules and beneficial antioxidant enzymes or other protective factors to recipient cells. By delivering antioxidants such as catalase and superoxide dismutase, EVs act as a cellular defense mechanism, helping restore redox balance and promoting tissue repair. This protective function is especially significant in degenerative and inflammatory diseases where oxidative stress is a major pathological driver. Chiaradia et al.’s work highlights both the mechanistic basis and the therapeutic promise of EVs in counteracting oxidative damage. As shown in Figure 6, this cluster is intimately linked to inflammation and angiogenesis, as EVs released under oxidative stress serve as signaling mediators to resolve tissue damage.

#### 3.4.2. Cluster #1 Small Extracellular Vesicles

This cluster is dedicated to the study of small (sEVs), which include exosomes. These nanosized vesicles are highly effective in delivering therapeutic cargo due to their ability to transfer functional proteins, lipids, and nucleic acids to target cells. sEVs are considered a promising “cell-free” therapy as they can replicate the regenerative effects of their parent cells without the risks associated with live-cell transplantation, such as immunogenicity or tumorigenic potential. Their unique properties make them ideal for drug delivery and modulating tissue repair. The highly cited and foundational paper in the study of sEVs, including exosomes, is the MISEV2018 guidelines paper by Théry et al. [14].

#### 3.4.3. Cluster #2 Mesenchymal Stromal Cells

This cluster focuses on MSCs as a primary source of therapeutic EVs. Cluster #2 represents a high-density thematic domain (Silhouette > 0.7) characterized by a significant citation burst beginning around 2017. The high total link strength between this cluster and Cluster #7 (Chronic Kidney Disease) indicates that MSC-derived EVs are the primary vehicle through which the field is testing organ-specific regeneration. The thematic dynamics show a transition from basic secretome identification toward the use of hydrogels for localized delivery, reflecting a maturation in the research pipeline. The therapeutic effects of MSCs in tissue repair and regeneration are largely mediated by their secretome, which includes EVs containing various anti-apoptotic, angiogenic, and anti-inflammatory factors. By using EVs from MSCs, researchers aim to harness these benefits in a safer and more scalable manner.

The highest cited work by Phinney, D. G., & Pittenger, M. F. (2017), discusses MSC-EVs as key mediators of the paracrine effects of MSCs, detailing their cargo of anti-apoptotic, angiogenic, and anti-inflammatory factors [18]. Bibliometric keywords in this cluster highlight a strong focus on wound healing and the development of hydrogel delivery systems to provide sustained release of EV cargo.

#### 3.4.4. Cluster #3 Muscle

This cluster is specific to the application of EVs in muscle regeneration and repair. EVs are crucial for intercellular communication in muscle tissue, helping to coordinate the response to injury and promote the regeneration of damaged myofibers. They can modulate immune responses, reduce inflammation, and stimulate the proliferation of satellite cells, which are essential for rebuilding muscle tissue after severe injury. The highest cited paper specifically addressing EVs in muscle regeneration and repair is a comprehensive 2017 review by Murphy et al. [19].

#### 3.4.5. Cluster #4 Mesenchymal Stem Cells

This cluster is broader than Cluster #2, focusing on the regenerative potential of MSCs themselves, with their paracrine signaling and EV secretion being the primary mechanisms of action. This research aims to understand how MSCs exert their effects and how this process can be manipulated to produce more potent EVs for a controlled, cell-free therapeutic approach.

The most highly cited paper on the regenerative potential of MSCs focusing on their paracrine signaling and EVs’ secretion is by M Kou et al., published in 2022 [20]. This domain specifically emphasizes the repair and proliferation of damaged tissue, as identified by the high-frequency keywords in the timeline analysis.

#### 3.4.6. Cluster #5 Extracellular Vesicles

This is a foundational cluster for the general topic of EVs. It includes research on EV biogenesis, their composition, and their fundamental role in intercellular communication. This cluster provides the basic scientific knowledge that underpins all other application-specific research, from tissue repair to drug delivery. The highest cited foundational review on EVs, covering their biogenesis, composition, and role in intercellular communication, is by Doyle and Wang (2019) [21].

#### 3.4.7. Cluster #6 Hotspots

Cluster #6 acts as the frontier zone of the network, showing the highest temporal activity in the 2022–2025 period. The analysis of bursting keywords—specifically “nanoparticles” and “targeted delivery”—deciphers a strategic shift where the research community is moving beyond natural EV biogenesis to resolve the translational barriers of biodistribution and potency. These include the use of engineered EVs for targeted drug delivery and the exploration of novel EV sources. Research is increasingly focused on modifying EVs to enhance their therapeutic potential, for example, by loading them with specific drugs or using genetic engineering to improve their targeting capabilities. This cluster points to the future of the field, where EVs are designed and optimized for specific clinical applications. Chen T et al. paper comprehensively discusses molecular engineering strategies for EVs, their therapeutic applications, and future directions up to 2024 [22]. Emerging hotspots involve combining EVs with nanoparticles for precision drug delivery and tissue repair.

#### 3.4.8. Cluster #7 Chronic Kidney Disease

The emergence of Cluster #7 (Chronic Kidney Disease) as a distinct research domain illustrates the field’s shift toward complex, multi-pathway pathologies. The focus here on regulating inflammation and fibrosis through exogenous EVs indicates a strategic move toward replacing invasive renal interventions with cell-free regenerative alternatives. The highest cited paper relevant to EVs therapy in CKD is a 2024 comprehensive review by Zheng et al. [23].

#### 3.4.9. Cluster #8 Bone Mesenchymal Stem Cells

This cluster is specific to the use of bone derived MSCs (BMSCs) and their EVs for bone regeneration. Research in this area investigates how BMSCs and their secreted vesicles can promote osteogenesis and heal bone fractures or defects. The highest cited paper specifically addressing BMSCs and their EVs for bone regeneration is a 2024 review by Wang et al. [24].

The emergence of specific clusters for Chronic Kidney Disease (Cluster #7) and Muscle (Cluster #3) indicates a strategic shift from general biological exploration to targeted therapeutic evidence, focusing on organs with high unmet regenerative needs.

#### 3.4.10. Conceptual Distinction Between MSC Clusters

While Cluster #2 (Mesenchymal Stromal Cells) and Cluster #4 (Mesenchymal Stem Cells) share a common biological origin, they are bibliometrically distinct based on their co-citation patterns and temporal evolution. Cluster #2 (Mesenchymal Stromal Cells) is characterized by more recent literature focusing on the “secretome” and the immunomodulatory properties of these cells in tissue repair. Cluster #4 (Mesenchymal Stem Cells) represents a broader, more foundational body of work centered on the regenerative potential and paracrine signaling mechanisms that established the transition from cell-based to cell-free therapy. The distinction between “Stromal” and “Stem” terminology in these clusters also reflects a shift in nomenclature within the International Society for Cell & Gene Therapy (ISCT) guidelines, where Cluster #2 aligns more closely with contemporary standardized reporting.

## 4. Discussion and Translational Outlook

**Figure 7:**
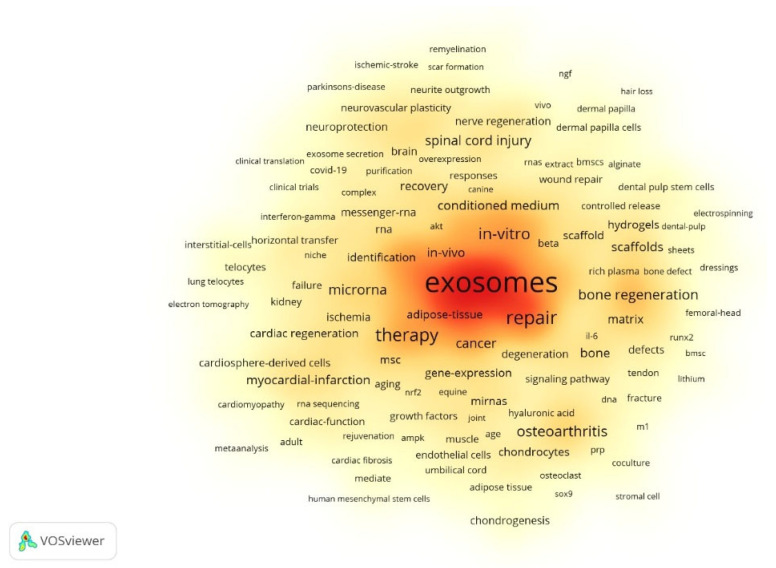
Keyword head map of extracellular vesicle-based regenerative medicine. Warmer colors (yellow to red) indicate higher keyword frequency and research intensity, highlighting core themes such as exosomes, therapy, repair, in vitro/in vivo studies.

The scientometric analysis from 2014 to 2024 reveals a notable progression in EV-based regenerative medicine, moving from foundational basic research towards more focused therapeutic applications. The domains of usage of EVs in regenerative medicine have expanded at a massive scale, as shown in the keyword heat map Figure 7. This maturation is supported by a robust scientific base, yet significant translational challenges remain. The dominance of China in publication volume and the USA in citation impact reflects distinct regional research strategies. China’s leading output is likely driven by massive, targeted government investment in regenerative medicine and “cell-free” biotechnology over the last decade. Conversely, the USA’s high H-index and citation dominance suggest a focus on “pioneering” foundational research—such as early EV biogenesis studies—which continues to serve as the intellectual bedrock for the field.

### 4.1. Interpretation of the Research Landscape

The bibliometric data reveals a field in a state of “functional transition.” The dominance of clusters focused on Oxidative Stress (Cluster #0) and Mesenchymal Stromal Cells (Cluster #2) indicates that the initial “discovery phase” of EV biology—focused on biogenesis and cargo—has reached saturation. The emergence of organ-specific clusters, such as Chronic Kidney Disease (Cluster #7) and Bone (Cluster #8), deciphered through co-citation networks, signifies that research is now “branching” into clinical niches where cell-free therapies offer the highest competitive advantage over traditional transplants. This shift from general vesicle biology to targeted therapeutic application is the primary driver behind the current publication trends.

### 4.2. Dynamics of Global Collaboration

The dominance of the China–USA–Korea axis in the cooperation network is not merely quantitative but reflects strategic resource sharing. The USA’s leadership in citation impact is driven by its role in establishing “Gold Standard” protocols, such as the MISEV guidelines. Meanwhile, the rapid surge in Chinese publications indicates a massive domestic push for “cell-free” regenerative medicine, likely fueled by national biotechnology initiatives aimed at overcoming the safety risks of live-cell transplantation. This suggests that international cooperation is currently driven by the need to harmonize these high-volume research outputs with global regulatory standards.

### 4.3. Standardization Issues

One of the foremost barriers is the lack of standardized protocols for EV isolation, quantification, and characterization. Without consensus on these standards, comparisons across laboratories become difficult, impairing reproducibility and slowing clinical advancement. Efforts like the MISEV guidelines provide some framework, but harmonizing these protocols globally remains a work in progress [25,26].

### 4.4. Scalability and Mass Production

Another key hurdle is scalable and cost-effective manufacturing of clinical-grade EVs. Current EV production techniques yield limited quantities, with batch-to-batch variability complicating quality control. Good Manufacturing Practice (GMP) adherence is needed to ensure consistent content, potency, and safety, yet many facilities lack the infrastructure essential for industrial-scale production. Developing reliable scale-up methods and quality assurance protocols is vital to transitioning EV therapies from bench to bedside [27,28]. A primary hurdle in the clinical transition of EV-based therapies is the transition from laboratory-scale isolation to industrial-scale GMP compliance. Current challenges include the need for standardized bioreactor systems to ensure batch-to-batch consistency in EV concentration and molecular cargo. Without established GMP-validated protocols for large-scale production, the cost-effectiveness and potency of clinical-grade EVs remain inconsistent.

### 4.5. Biodistribution and Pharmacokinetics

Understanding the in vivo biodistribution and pharmacokinetics of EVs is crucial for therapeutic targeting and efficacy. Studies show EVs have rapid plasma clearance and tend to accumulate in organs like the liver and spleen, which act as biological barriers. Their short half-life and varied tissue uptake require innovative engineering approaches to improve delivery and retention at target sites. However, more detailed mechanistic insights into EV trafficking and fate at the cellular and molecular levels remain necessary [29,30].

### 4.6. Regulatory Challenges

The regulatory landscape is evolving, addressing EV heterogeneity, classification, and quality attributes. Regulatory bodies emphasize the need for stringent potency assays and standardized characterization to ensure safety and efficacy. Collaboration between academic, industrial, and regulatory stakeholders is essential to develop clear pathways for EV product approval [31,32]. The classification of EV-based products within the Advanced Therapy Medicinal Product (ATMP) framework is a major regulatory ambiguity. Because EVs are often viewed as biological derivatives rather than whole cells, they occupy a unique regulatory “gray area” regarding safety and potency assays. Collaboration between academic and industrial stakeholders is required to define these products under current ATMP standards [33] to ensure a clear pathway for human application and commercialization.

### 4.7. Shift to Therapeutic Focus and Thematic Clustering

Research increasingly prioritizes application-specific studies, highlighting CKD and muscle regeneration as major thematic clusters. Engineered EVs with enhanced immunomodulatory and regenerative properties demonstrate promising therapeutic effects in preclinical CKD models. This targeted focus evidences concerted efforts to translate foundational science into clinically practical treatments [33].

### 4.8. Limitations

While this study provides a comprehensive overview of the research landscape, it is not without limitations. First, data were retrieved exclusively from the WoS Core Collection. Although WoS is considered the most reliable database for scientometric analysis due to its rigorous indexing and detailed citation metadata, it may not capture every relevant study indexed in other databases such as Scopus or PubMed. While the current approach ensured methodological consistency, future bibliometric analyses may integrate Scopus and PubMed to broaden coverage and validate cross-database trends. Consequently, some highly specialized or emerging publications in the field of EVs might be omitted from the quantitative metrics. Future studies could benefit from a multi-database approach to provide an even broader perspective on the field’s evolution.

## 5. Emerging Trends and Future Prospects

The future of EV-based regenerative medicine is characterized by a strategic shift towards enhancing and optimizing EVs for specific clinical applications, moving beyond their natural form.

*Engineering and Bio-fabrication*: Researchers are increasingly focused on engineering EVs to improve their therapeutic potential. This involves modifying the EV membrane to enhance targeting capabilities, for example, by adding cell-specific ligands or peptides to increase their accumulation in desired tissues [34,35]. EVs are also being loaded with specific therapeutic cargo, such as drugs, small interfering RNAs (siRNA), or even CRISPR/Cas9 systems, to achieve a more potent and targeted therapeutic effect.

*Novel Sources*: While MSCs are a dominant source, there is growing interest in EVs from other cell types, including induced pluripotent stem cells (iPSCs), macrophages, and even non-mammalian sources [36]. This diversification of sources is driven by a desire for better safety profiles, scalability, and unique therapeutic benefits. For instance, certain sources might yield EVs with a distinct miRNA signature that could be more effective for a particular condition [37].

*Standardization and Regulation*: As the field progresses toward clinical trials, there is a strong push for global standardization of EV isolation, characterization, and quality control protocols. This is crucial for ensuring the safety, purity, and consistent potency of EV products for human use and is necessary to satisfy regulatory requirements. The development of standardized guidelines, such as the MISEV guidelines, is a key step in this process [38].

*Clinical Translation*: The number of registered clinical trials for EV-based therapies is steadily increasing, particularly for conditions like CDK and musculoskeletal injuries [39]. This trend signifies the critical move from preclinical studies to human application. However, challenges remain regarding optimal dosing, administration routes, and understanding how EV therapies will be regulated and approved. Research into biodistribution and pharmacokinetics is also vital to ensure that EVs reach their intended targets effectively and safely [39]. The ultimate goal is to offer a holistic, cell-free approach that not only regenerates tissue but also addresses underlying pathologies like endothelial dysfunction [23].

The future of EV-based therapy in regenerative medicine is bright, but its widespread clinical use depends on overcoming several key challenges. Researchers are modifying the EV membrane to improve their ability to reach specific tissues and loading them with therapeutic cargo like drugs, genes, or other bioactive molecules to enhance their healing capabilities [40].

In addition to engineering, these alternative sources, such as iPSCs, macrophages, and even plant-based materials, could offer advantages in terms of safety, scalability, and unique therapeutic benefits [41,42,43].

The ultimate goal is to move from basic science to clinical application, with ongoing research into the biodistribution and pharmacokinetics of EVs. The field is poised to offer a cell-free therapeutic approach that not only helps regenerate tissue but also addresses underlying pathologies like inflammation and oxidative stress [44].

## 6. Conclusions

The bibliometric analysis of EV-based regenerative medicine (2014–2025) deciphers a field transitioning from a “discovery-centric” era to a “translation-centric” era. The high silhouette values of clusters focusing on Chronic Kidney Disease (Cluster #7) and Bone (Cluster #8) provide quantitative evidence that the field has successfully identified its primary clinical niches.

Strategically, the work highlights three critical pivots for the research community: Standardization is the intellectual core: The central co-citation of MISEV guidelines confirms that methodological rigor is the prerequisite for all downstream therapeutic success. Technological convergence: The emerging “Hotspots” (Cluster #6) identify a necessary merger between EV biology and bio-engineering, moving toward “designer EVs” to resolve the current bottlenecks of rapid plasma clearance and off-target accumulation. Global synergy: While publication volume is geographically concentrated in China, the USA remains the engine of foundational impact (H-index), suggesting that future breakthroughs depend on harmonizing high-volume production with standardized, high-impact safety protocols.

Overcoming the identified regulatory and GMP manufacturing hurdles is the final requirement to translate these bibliometric trends into a reproducible cell-free reality for regenerative medicine.

## Figures and Tables

**Table 1:** Top journals publishing extracellular vesicle based regenerative medicine research works.

Journal Rank	Journal	Documents	Citations	Total Link Strength
1	*Stem Cell Research & Therapy*	134	7613	1159
2	*International Journal of Molecular Sciences*	121	3713	660
3	*Biomaterials*	53	5203	588
4	*Stem Cells Translational Medicine*	35	4170	488
5	*Theranostics*	35	4433	488
6	*Scientific Reports*	59	3044	382
7	*Acta Biomaterialia*	34	1792	309
8	*Journal of Nanobiotechnology*	39	1794	309
9	*Bioactive Materials*	30	2167	295
10	*ACS Nano*	31	11,622	290
11	*Journal of Extracellular Vesicles*	25	4119	311
12	*Stem Cell Reviews and Reports*	31	1149	311
13	*Stem Cells International*	30	1168	295
14	*International Journal of Nanomedicine*	28	1125	284
15	*Frontiers in Bioengineering and Biotechnology*	33	1045	284
16	*Cells*	24	934	284
17	*ACS Applied Materials & Interfaces*	20	1345	284

**Table 2:** Leading Contributors in EV-based Regenerative Medicine (2014–2024).

Category	Top Entities	Key Metrics/Influence
Authors	C. Théry, R.C. Lai, B. Zhang, S. Bruno	Théry and Lai lead with 658 and 509 citations respectively.
Institutions	Univ. of California System, Harvard Univ.	Key drivers of high-impact foundational research.
Countries	China (Volume), USA (Impact)	China ranks 1st in publications; USA leads in H-index and citations.

## Data Availability

The data generated from this study within the manuscript/Supplementary Files.

## Supplementary Materials

The Supplementary Materials contain original and additional figures, tables, and supporting data that complement the findings of this study. The following supporting information can be downloaded at: https://www.mdpi.com/article/10.3390/bioengineering13020247/s1.
