# Supplementary figures and images for: Global Research Trends in Extracellular Vesicle–Based Therapy for Regenerative Medicine: A Bibliometric Analysis (2014–2024)
Source: Bioengineering (Basel). 2026 Feb 20;13(2):247. doi: 10.3390/bioengineering13020247 (PMC12937803; doi:10.3390/bioengineering13020247)

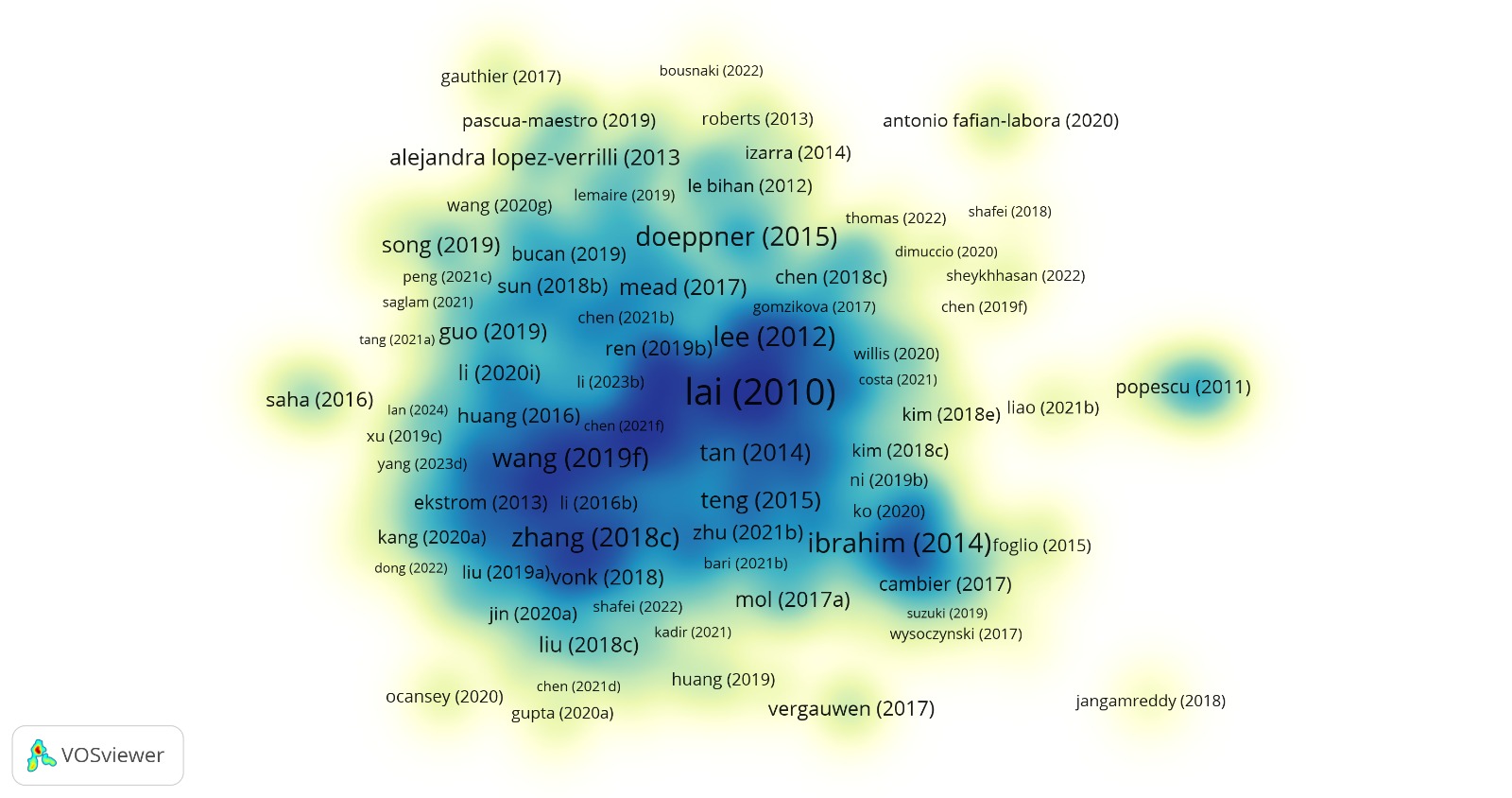

Supplement: Supplementary file 1 [file bioengineering-13-00247-s001.zip › download_(1).jpg]

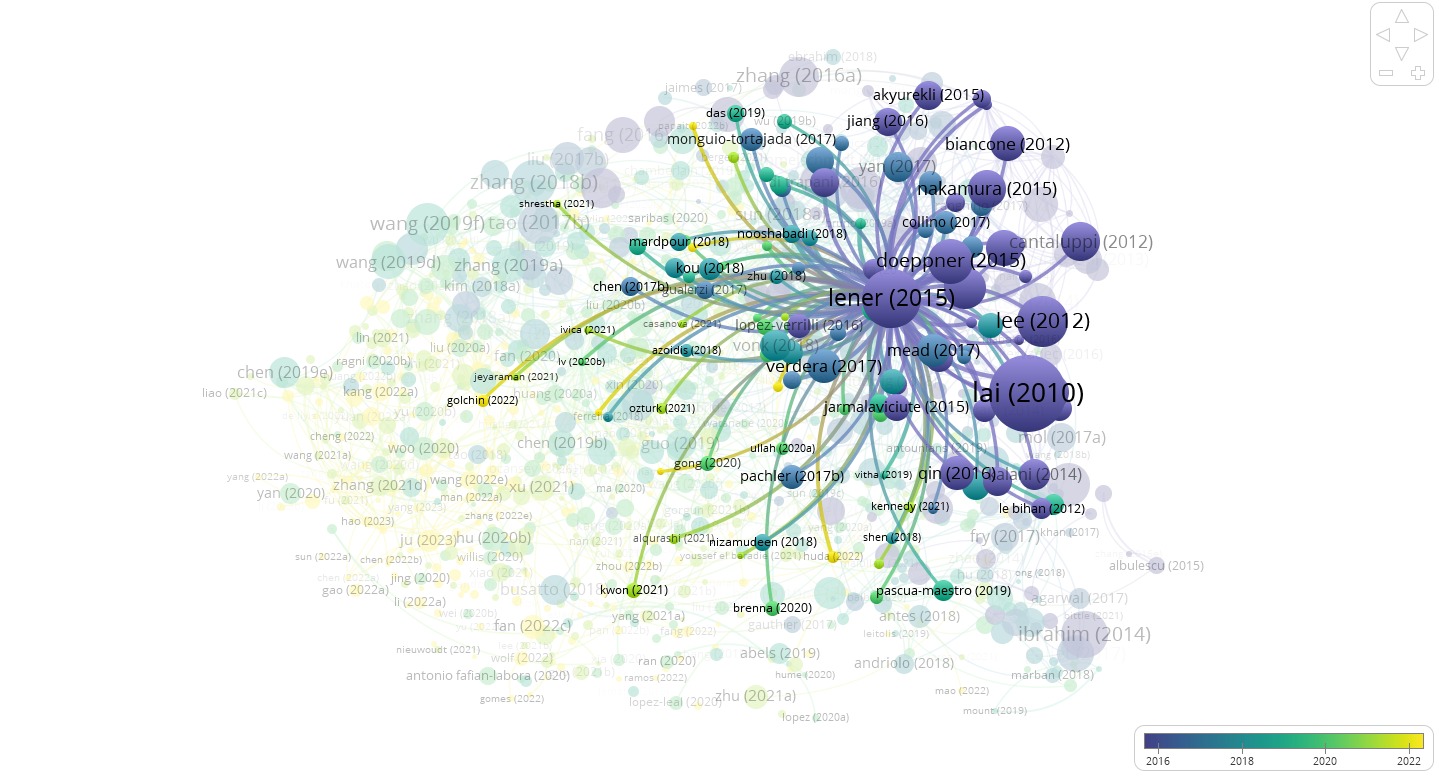

Supplement: Supplementary file 1 [file bioengineering-13-00247-s001.zip › download_(2).jpg]

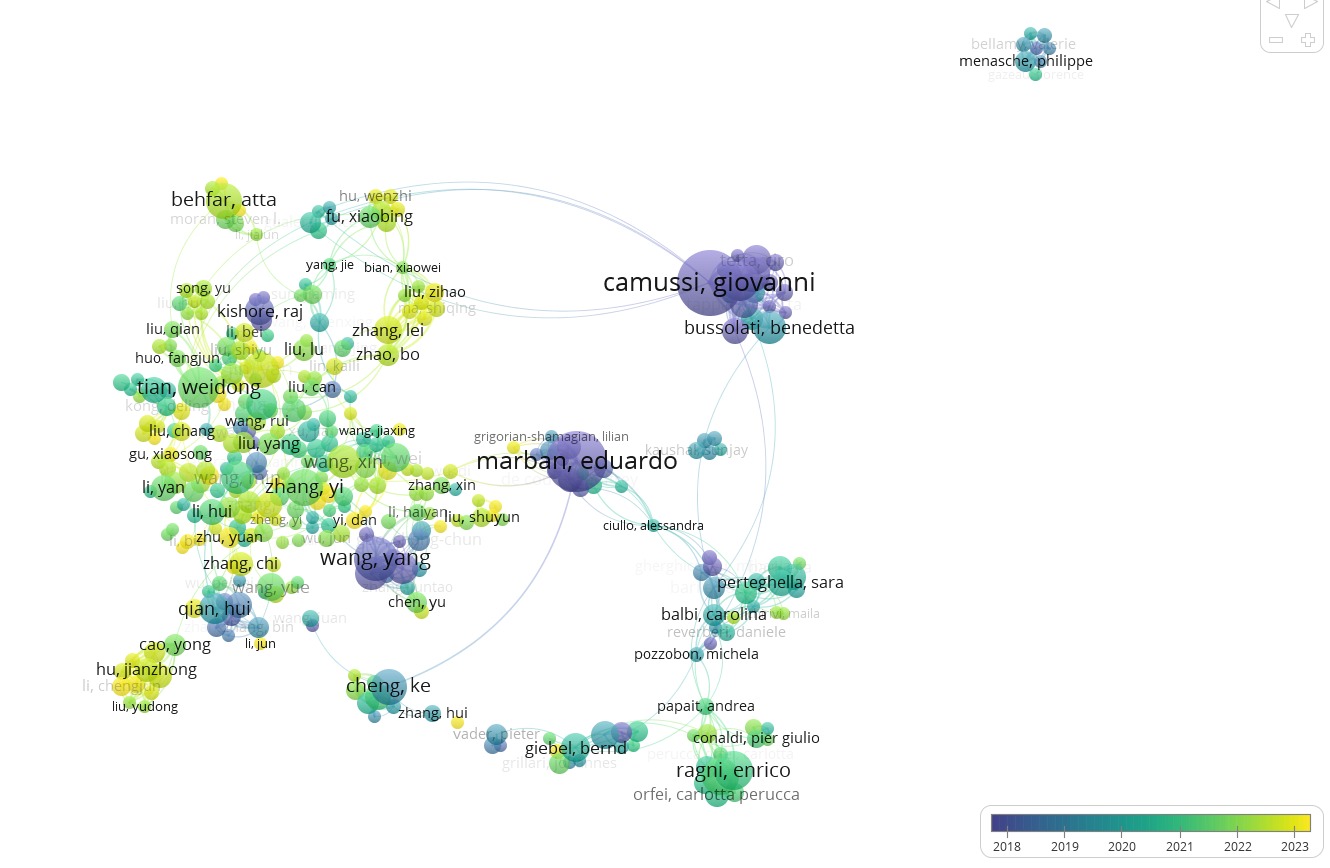

Supplement: Supplementary file 1 [file bioengineering-13-00247-s001.zip › download_(3).jpg]

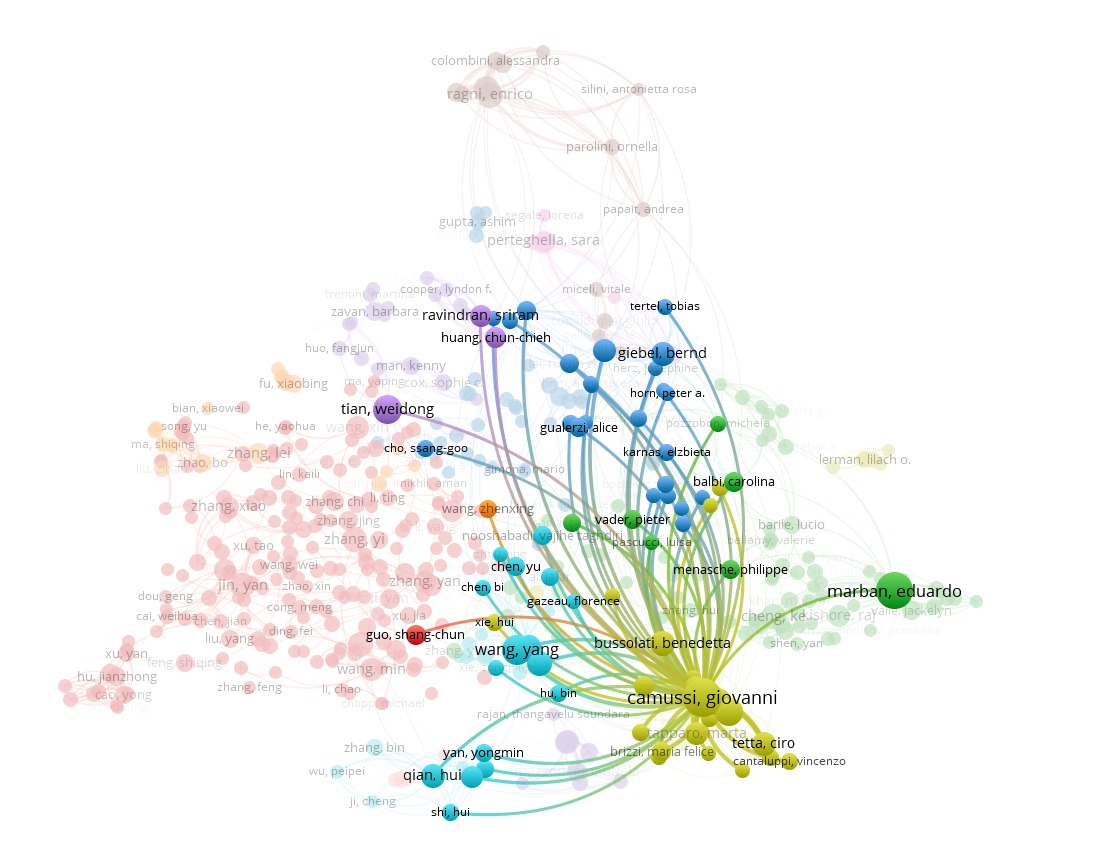

Supplement: Supplementary file 1 [file bioengineering-13-00247-s001.zip › download_(4).jpg]

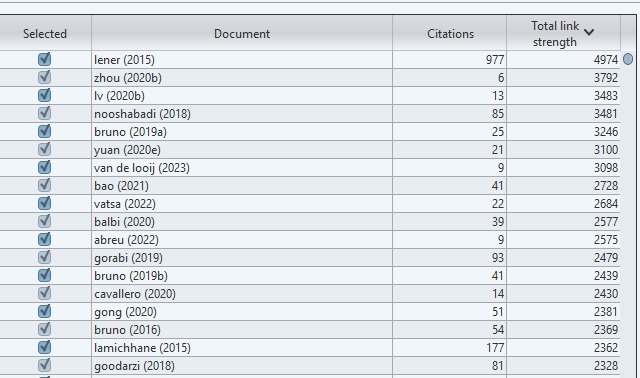

Supplement: Supplementary file 1 [file bioengineering-13-00247-s001.zip › download_(5).jpg]

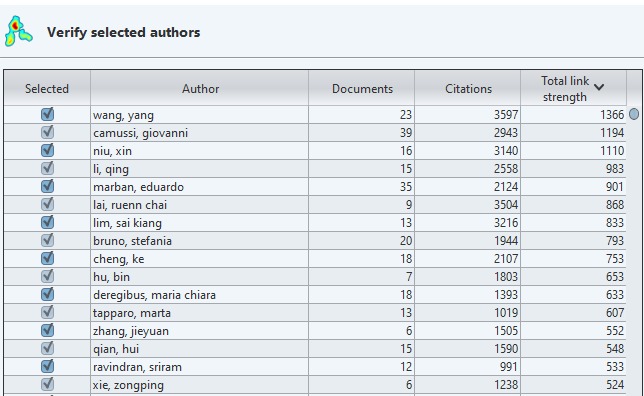

Supplement: Supplementary file 1 [file bioengineering-13-00247-s001.zip › download_(6).jpg]

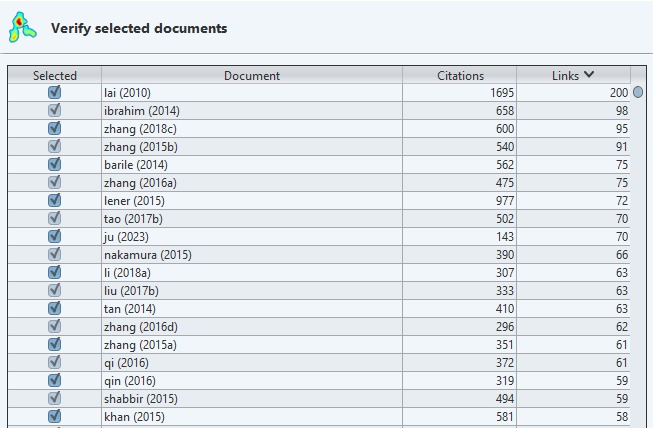

Supplement: Supplementary file 1 [file bioengineering-13-00247-s001.zip › download_(7).jpg]

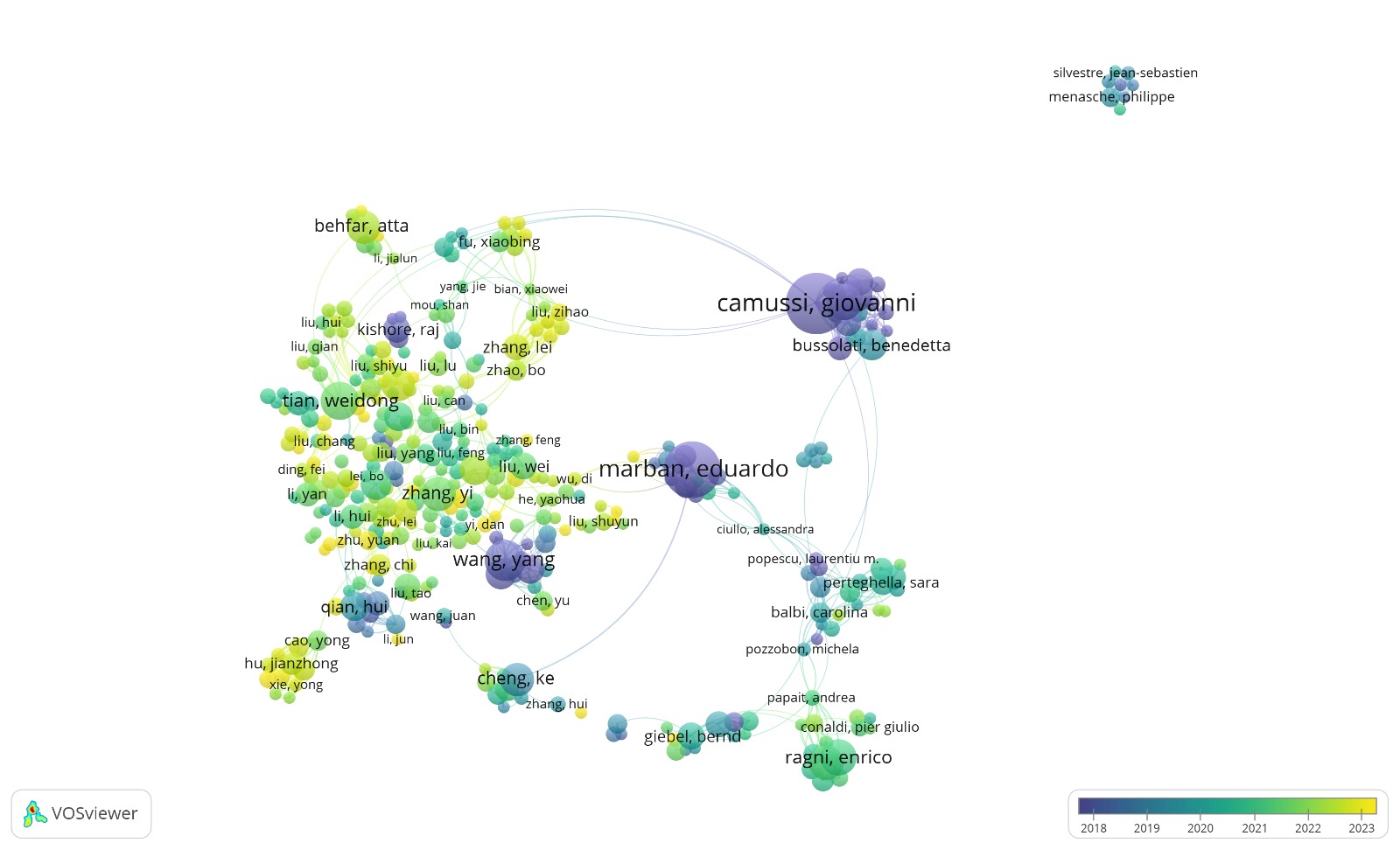

Supplement: Supplementary file 1 [file bioengineering-13-00247-s001.zip › download_(8).jpg]

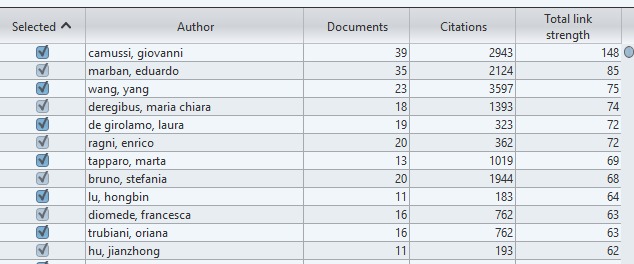

Supplement: Supplementary file 1 [file bioengineering-13-00247-s001.zip › download_(9).jpg]

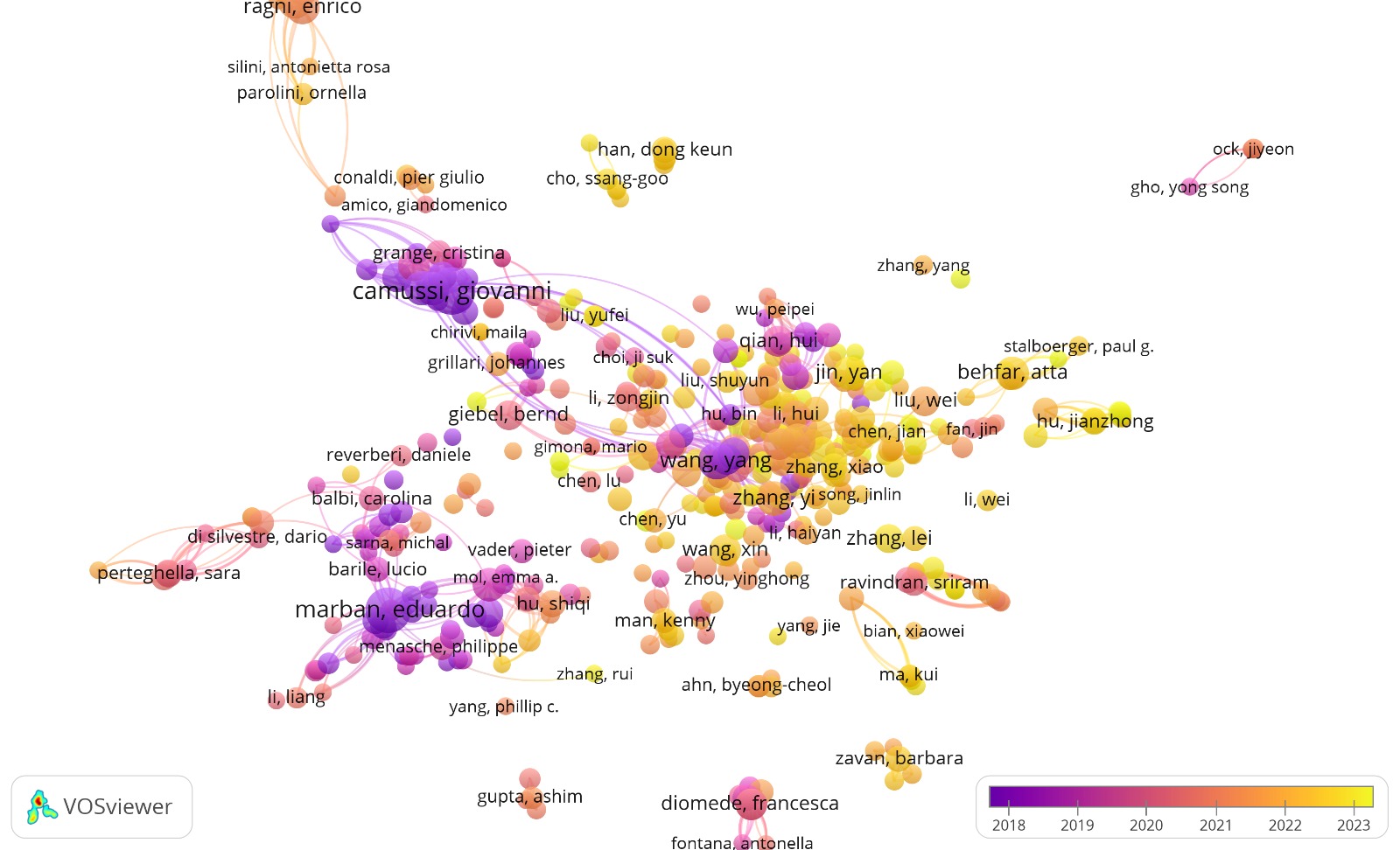

Supplement: Supplementary file 1 [file bioengineering-13-00247-s001.zip › download_(10).jpg]

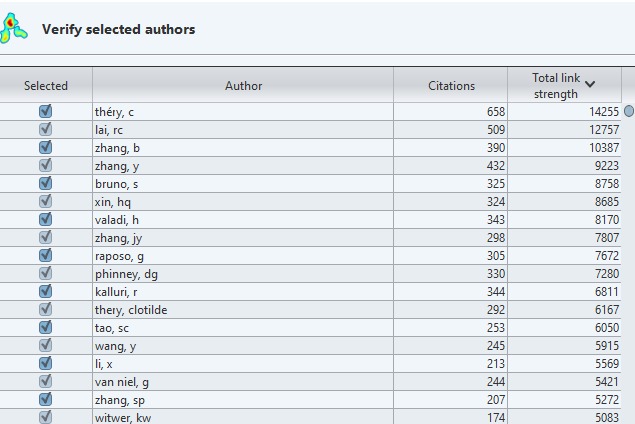

Supplement: Supplementary file 1 [file bioengineering-13-00247-s001.zip › download_(11).jpg]

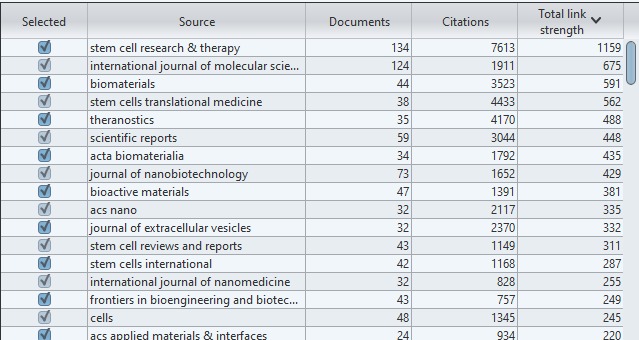

Supplement: Supplementary file 1 [file bioengineering-13-00247-s001.zip › download_(12).jpg]

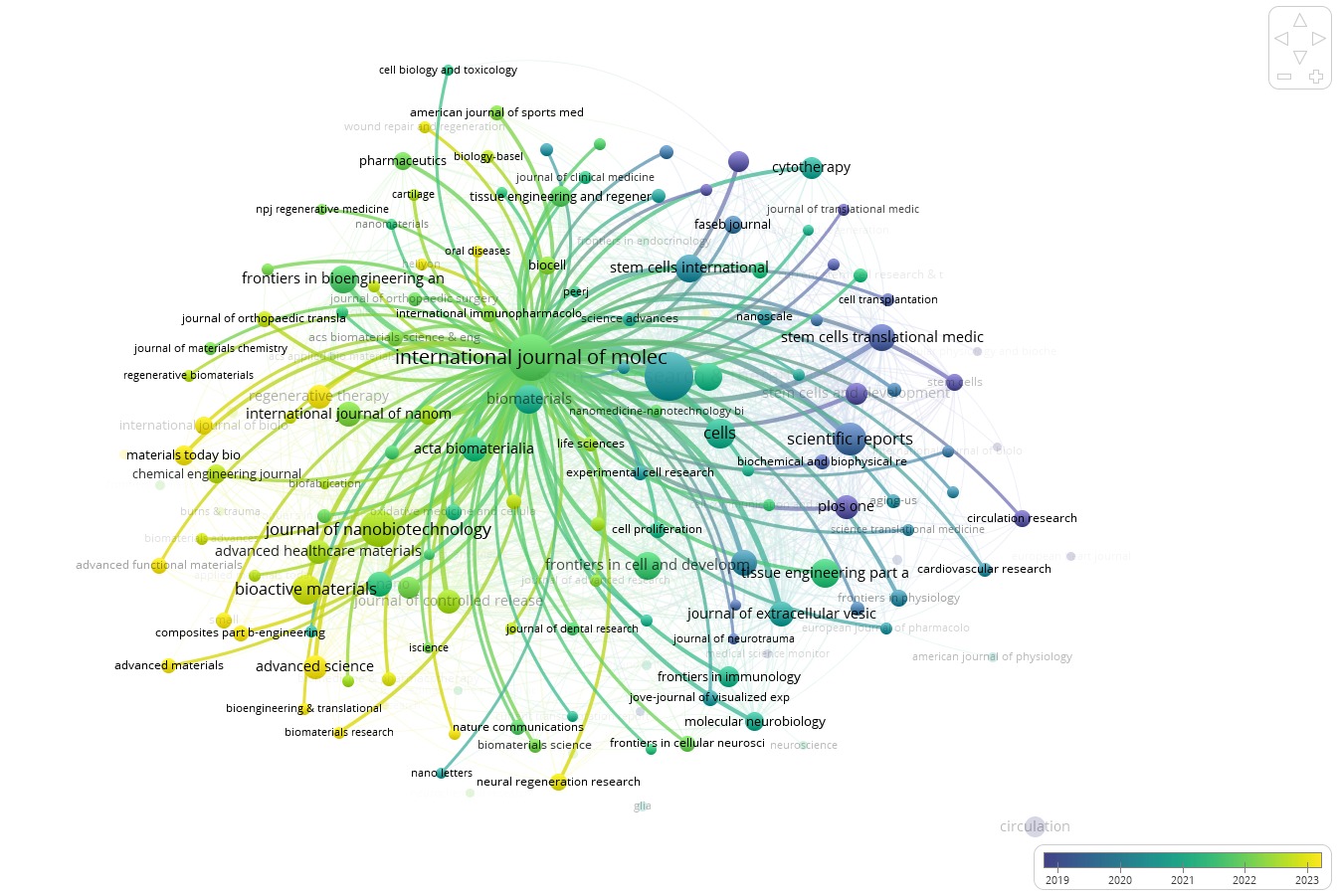

Supplement: Supplementary file 1 [file bioengineering-13-00247-s001.zip › download_(13).jpg]

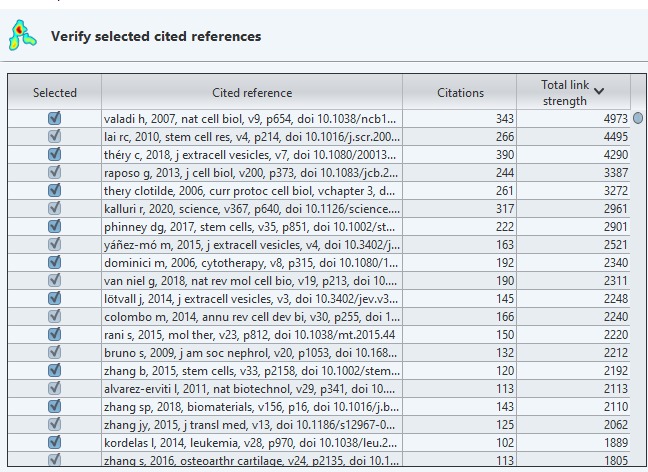

Supplement: Supplementary file 1 [file bioengineering-13-00247-s001.zip › download_(14).jpg]

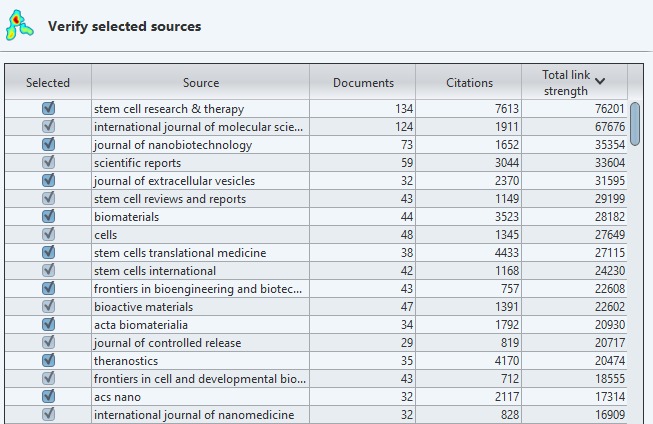

Supplement: Supplementary file 1 [file bioengineering-13-00247-s001.zip › download_(15).jpg]

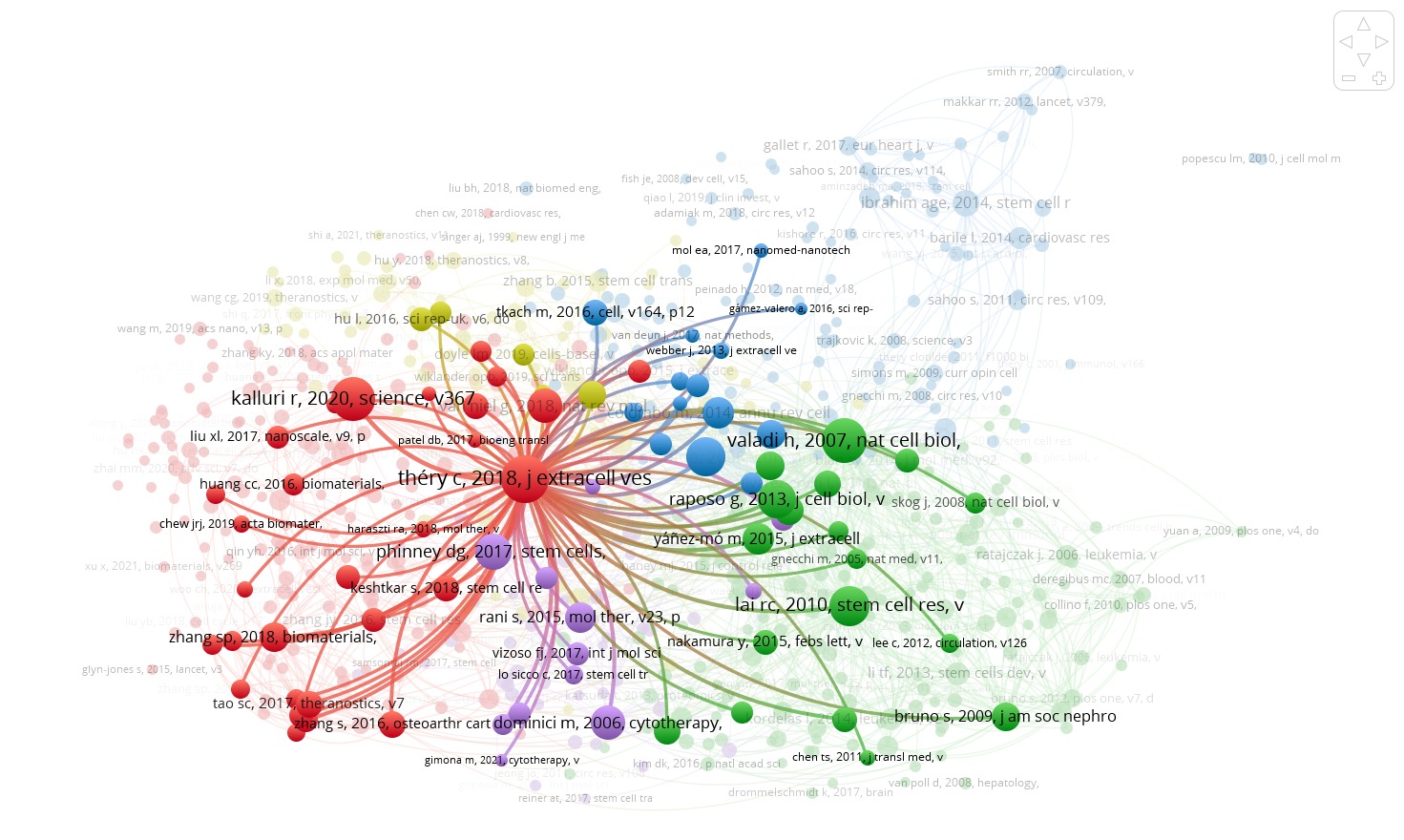

Supplement: Supplementary file 1 [file bioengineering-13-00247-s001.zip › download_(16).jpg]

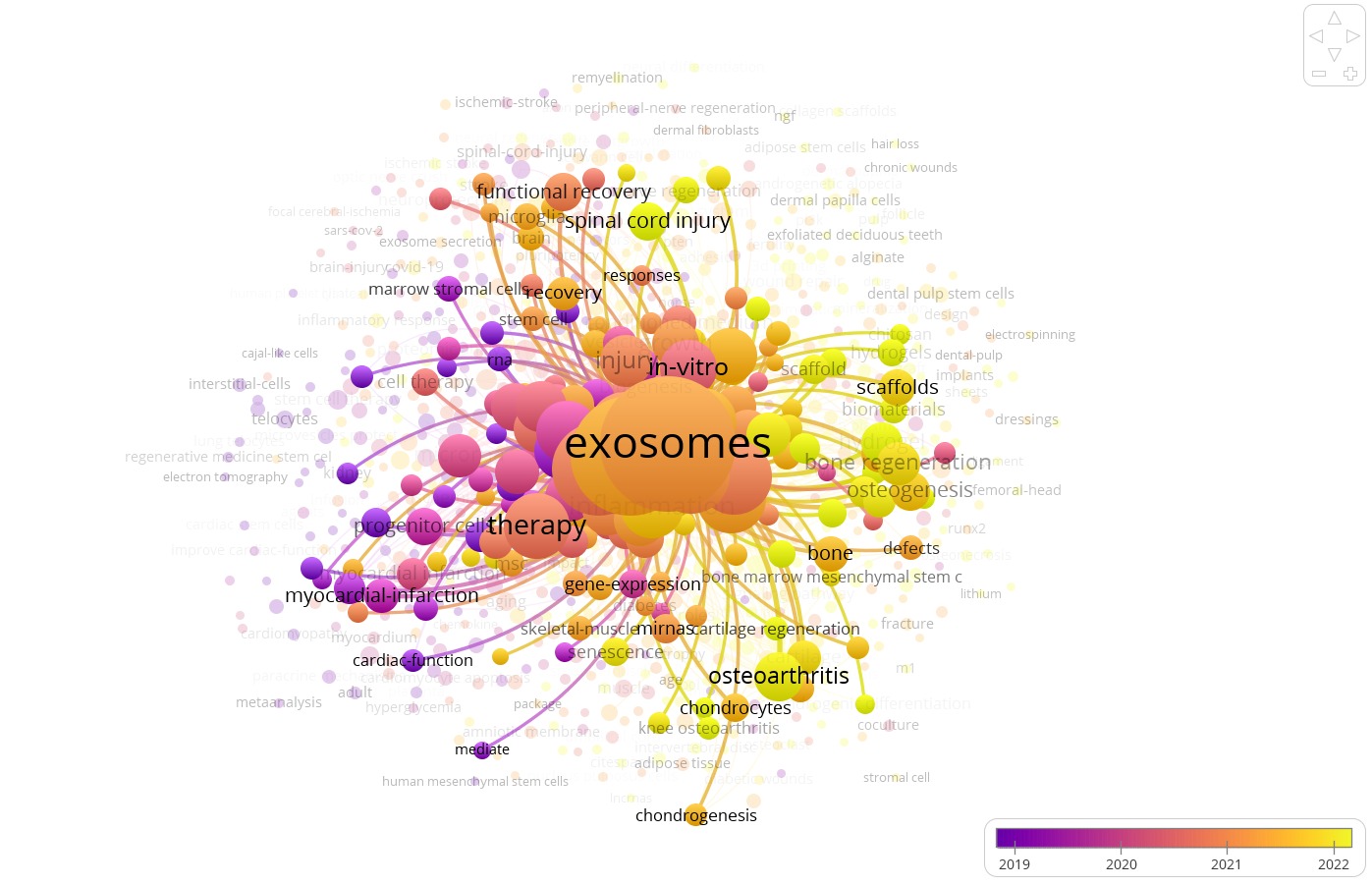

Supplement: Supplementary file 1 [file bioengineering-13-00247-s001.zip › download_(17).jpg]

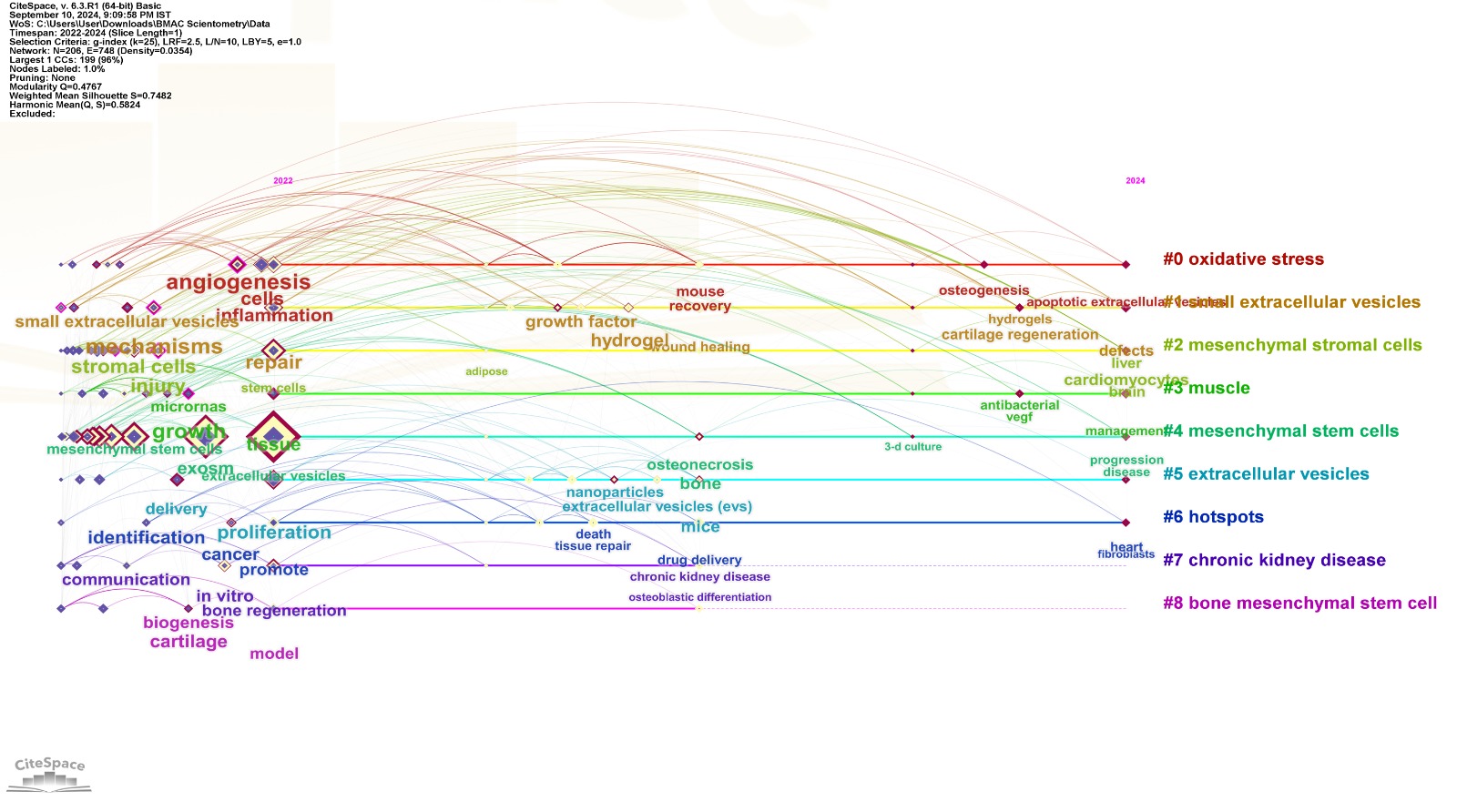

Supplement: Supplementary file 1 [file bioengineering-13-00247-s001.zip › download_(18).jpg]

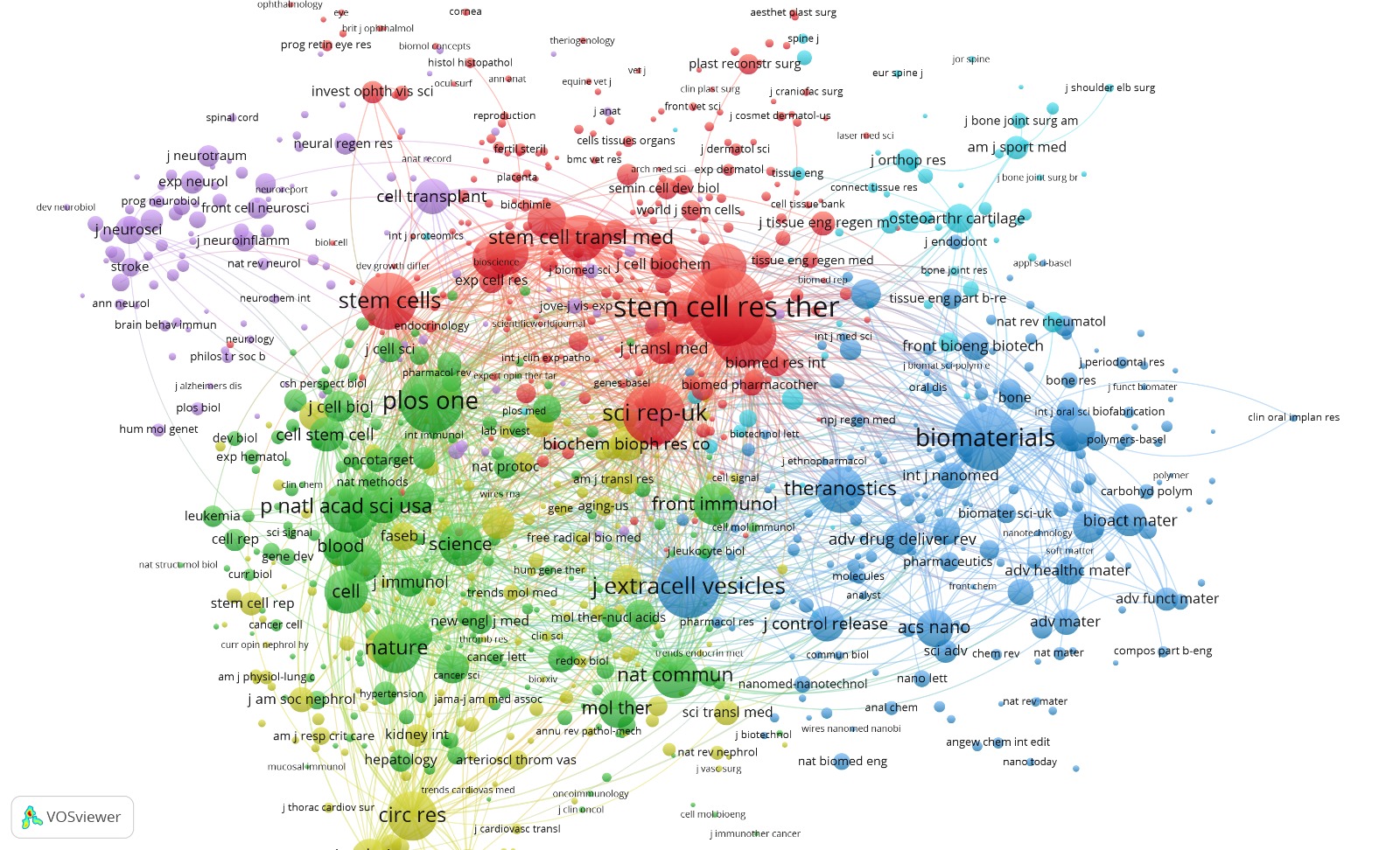

Supplement: Supplementary file 1 [file bioengineering-13-00247-s001.zip › download_(19).jpg]

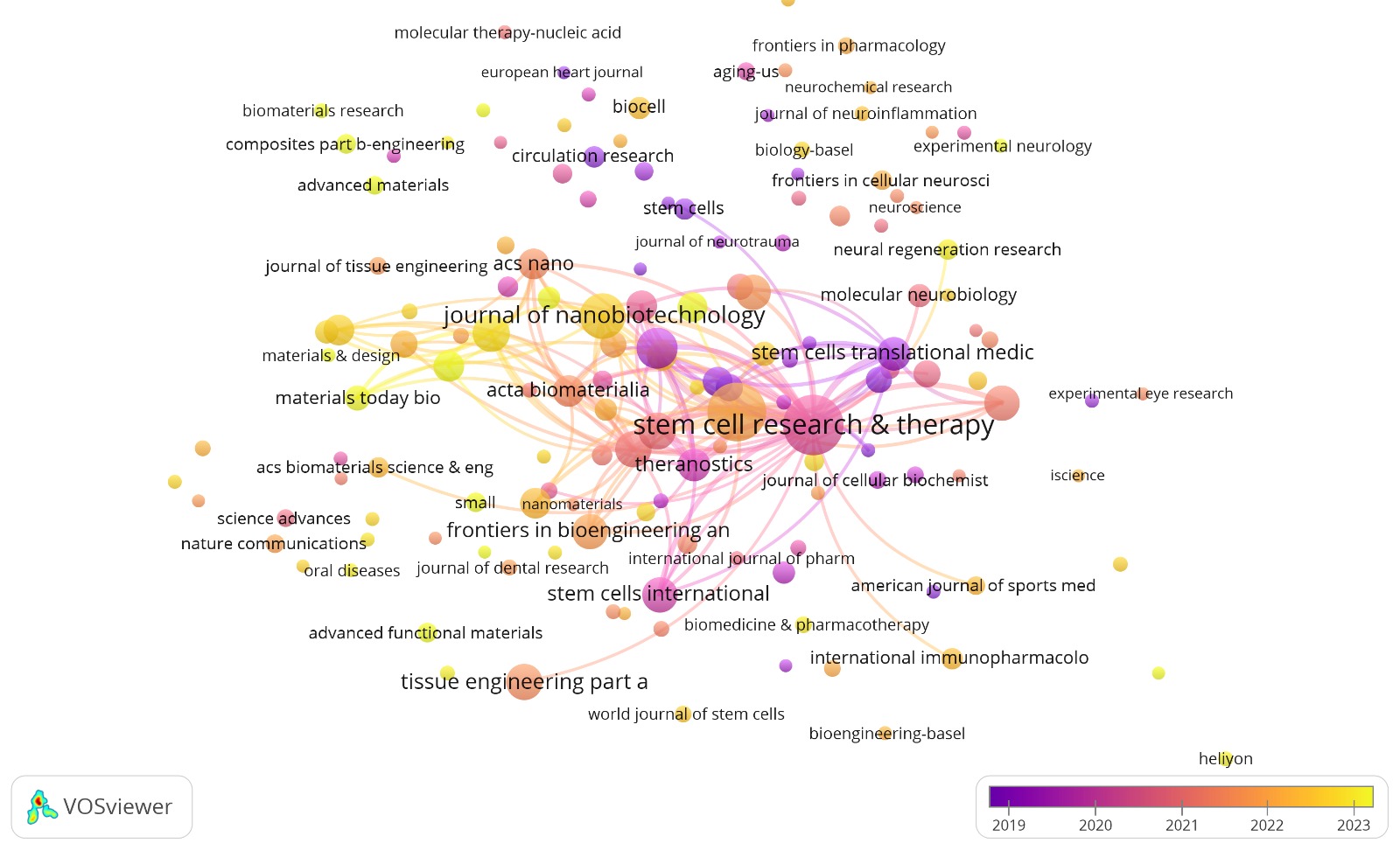

Supplement: Supplementary file 1 [file bioengineering-13-00247-s001.zip › download_(20).jpg]

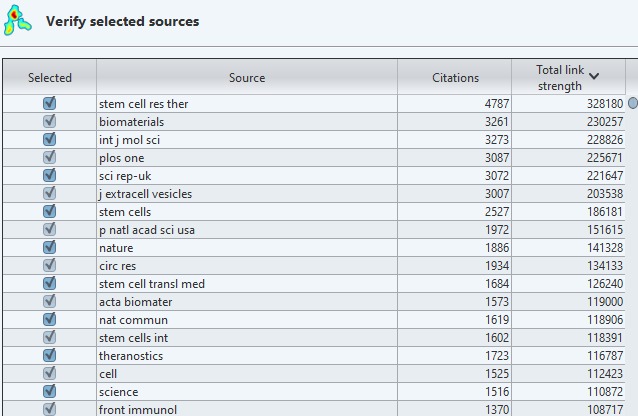

Supplement: Supplementary file 1 [file bioengineering-13-00247-s001.zip › download_(21).jpg]

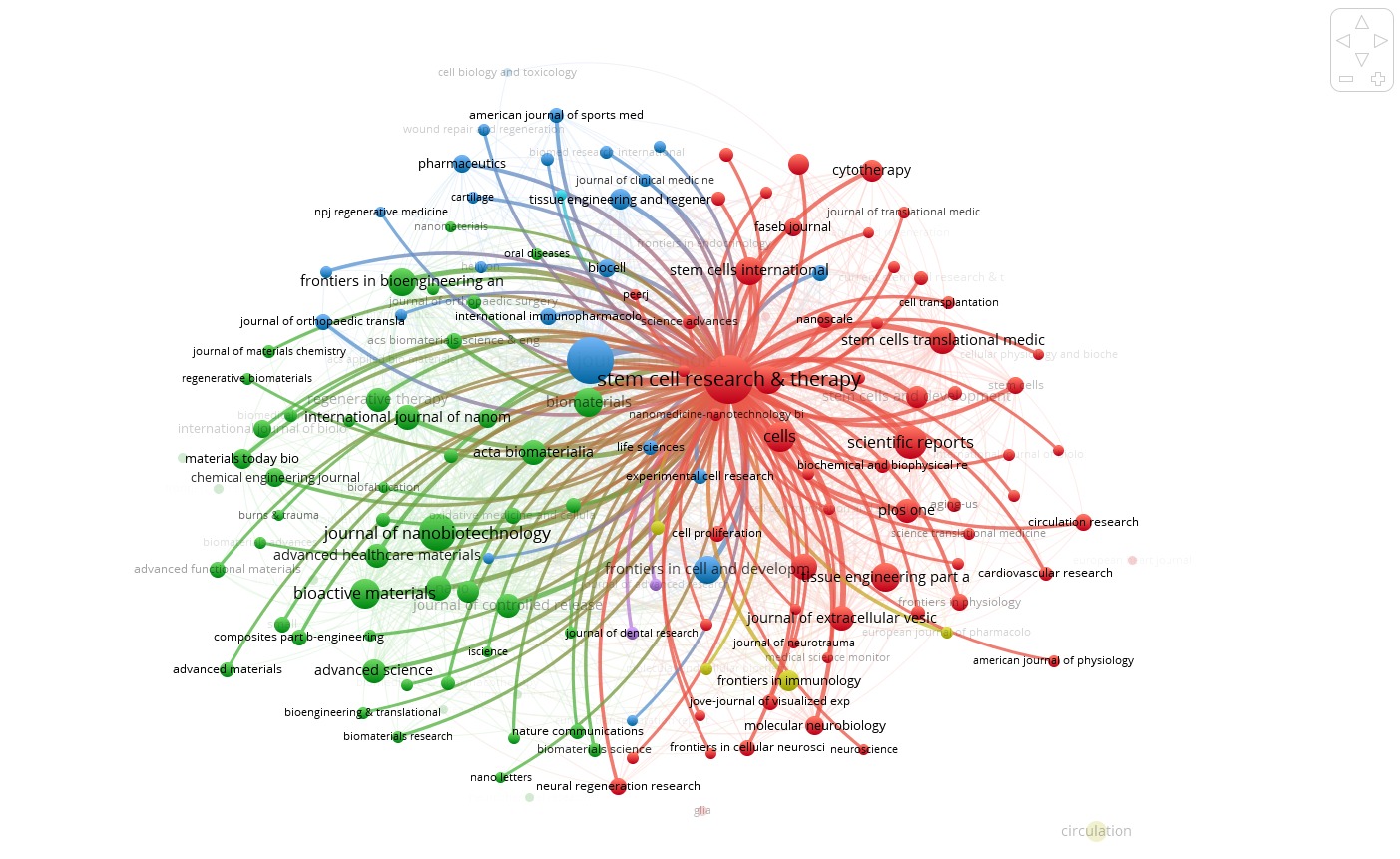

Supplement: Supplementary file 1 [file bioengineering-13-00247-s001.zip › download_(22).jpg]

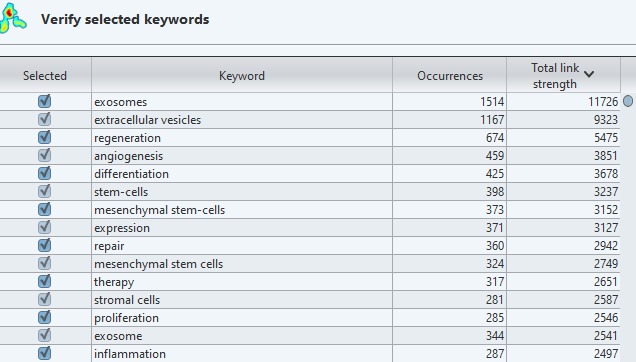

Supplement: Supplementary file 1 [file bioengineering-13-00247-s001.zip › download_(23).jpg]

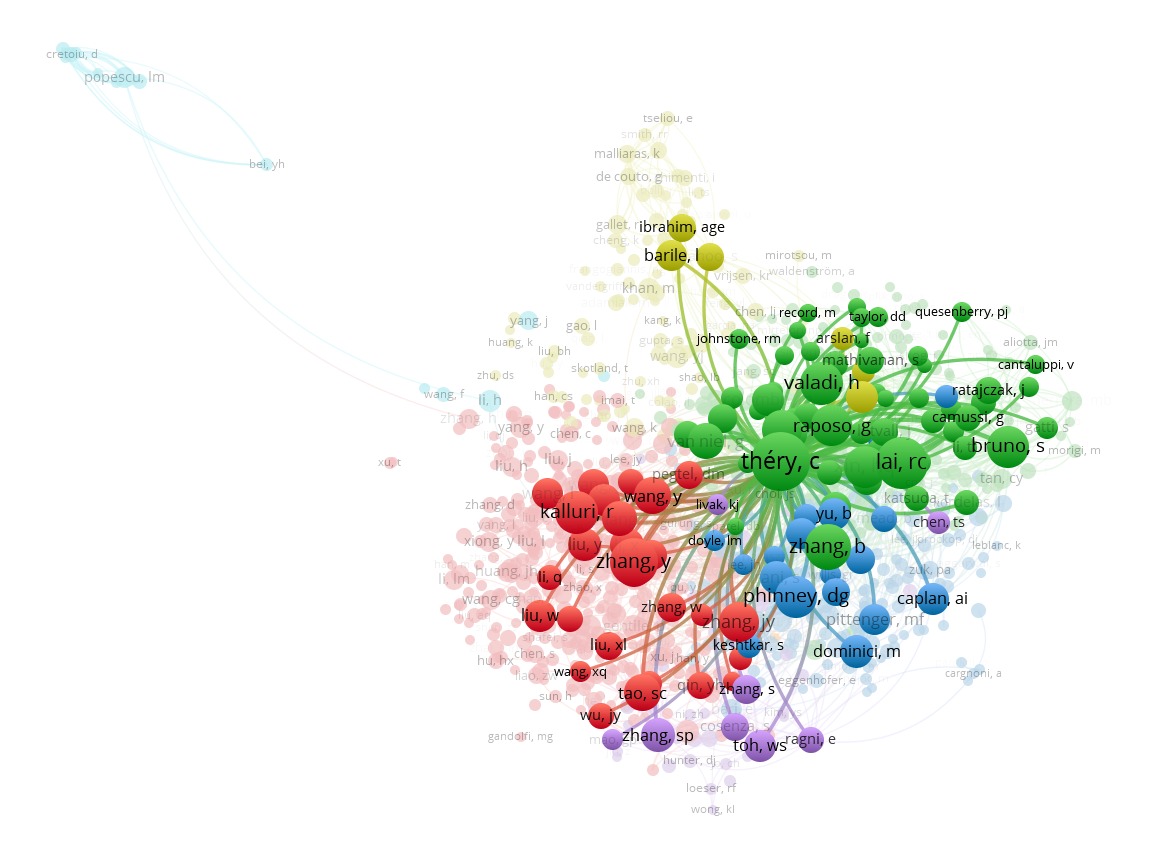

Supplement: Supplementary file 1 [file bioengineering-13-00247-s001.zip › download_(24).jpg]

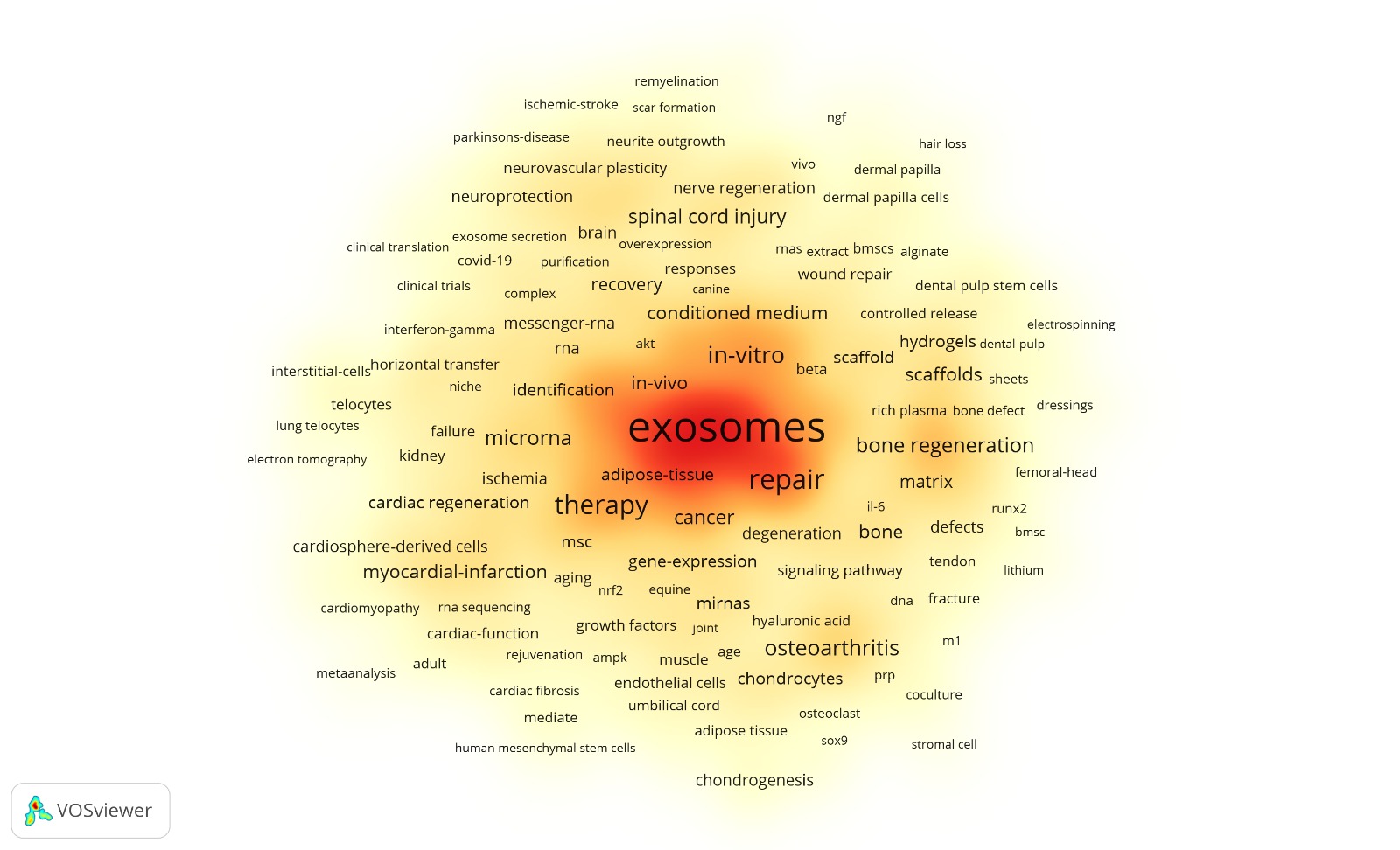

Supplement: Supplementary file 1 [file bioengineering-13-00247-s001.zip › download_(25).jpg]
